# Quantitative proteomic screening uncovers candidate diagnostic and monitoring serum biomarkers of ankylosing spondylitis

**DOI:** 10.1186/s13075-023-03044-4

**Published:** 2023-04-11

**Authors:** Mark Hwang, Shervin Assassi, Jim Zheng, Jessica Castillo, Reyna Chavez, Kamala Vanarsa, Chandra Mohan, John Reveille

**Affiliations:** 1 McGovern Medical School, University of Texas Health Science Center at Houston, 6431 Fannin MSB.5270, TX 77030 Houston, USA; 2 School of Biomedical Informatics, UTHealth Houston, Houston, TX USA; 3 Biomedical Engineering, University of Houston, Houston, TX USA

**Keywords:** Ankylosing spondylitis, Axial spondyloarthritis, Proteomics, Biomarkers, Diagnosis, Disease activity

## Abstract

**Background:**

We sought to discover serum biomarkers of ankylosing spondylitis (AS) for diagnosis and monitoring disease activity.

**Methods:**

We studied biologic-treatment-naïve AS and healthy control (HC) patients’ sera. Eighty samples matched by age, gender, and race (1:1:1 ratio) for AS patients with active disease, inactive disease, and HC were analyzed with SOMAscan™, an aptamer-based discovery platform. *T*-tests tests were performed for high/low-disease activity AS patients versus HCs (diagnosis) and high versus low disease activity (Monitoring) in a 2:1 and 1:1 ratio, respectively, to identify differentially expressed proteins (DEPs).

We used the Cytoscape Molecular Complex Detection (MCODE) plugin to find clusters in protein–protein interaction networks and Ingenuity Pathway Analysis (IPA) for upstream regulators. Lasso regression analysis was performed for diagnosis.

**Results:**

Of the 1317 proteins detected in our diagnosis and monitoring analyses, 367 and 167 (317 and 59, FDR-corrected *q* < .05) DEPs, respectively, were detected. MCODE identified complement, IL-10 signaling, and immune/interleukin signaling as the top 3 diagnosis PPI clusters. Complement, extracellular matrix organization/proteoglycans, and MAPK/RAS signaling were the top 3 monitoring PPI clusters. IPA showed interleukin 23/17 (interleukin 22, interleukin 23A), TNF (TNF receptor-associated factor 3), cGAS-STING (cyclic GMP-AMP synthase, Stimulator of Interferon Gene 1), and Jak/Stat (Signal transducer and activator of transcription 1), signaling in predicted upstream regulators. Lasso regression identified a Diagnostic 13-protein model predictive of AS. This model had a sensitivity of 0.75, specificity of 0.90, a kappa of 0.59, and overall accuracy of 0.80 (95% CI: 0.61–0.92). The AS vs HC ROC curve was 0.79 (95% CI: 0.61–0.96).

**Conclusion:**

We identified multiple candidate AS diagnostic and disease activity monitoring serum biomarkers using a comprehensive proteomic screen. Enrichment analysis identified key pathways in AS diagnosis and monitoring. Lasso regression identified a multi-protein panel with modest predictive ability.

**Supplementary Information:**

The online version contains supplementary material available at 10.1186/s13075-023-03044-4.

## Introduction

Ankylosing spondylitis (AS) is a chronic, systemic inflammatory disease with cardinal features of inflammatory back pain, sacroiliitis, and spinal fusion that lead to significant functional impairment [1]. AS is also associated with extra-articular features such as uveitis, psoriasis, and inflammatory bowel disease, which can precede or follow characteristic spinal involvement [2]. AS is a subset of axial spondyloarthritis (AxSpA), estimated to affect up to 2–3 million people in the USA [3]. Genetic studies in AS have identified over 113 susceptibility loci that have suggested pathologic mechanisms for this complex disease [4–6]. Gene expression studies of peripheral blood at both the bulk and single-cell level have recently provided insight into disease pathogenesis [7–9].

Large-scale examination of proteins, unlike DNA and RNA, remains scarce and limited in AS. Recent mass spectrometry-based studies of AS patients compared to healthy controls have revealed dysregulation of serum proteins—including complement, metalloproteinases, and serum amyloid A1 (SAA1) [10–12]. Dynamic range limitations of this lab technique however limit proteome coverage, which may restrict molecular characterization of AS [13].

Biomarkers have been defined as “a characteristic that is objectively measured and evaluated as an indicator of normal biological processes, pathogenic processes, or pharmacologic responses to a therapeutic intervention” [14]. Many rheumatic diseases now have incorporated serologic and proteomic markers into their classification criteria and disease activity measures. Biomarkers that can aid in the diagnosis and monitoring of disease activity of AS could improve clinical care and advance our pathophysiology understanding. The two most utilized in AS clinical practice and trials include C-reactive protein (CRP) and erythrocyte sedimentation rate (ESR). These lack sensitivity with two thirds of those with established AS and clinically active disease having within normal levels [15]. To this end, a great need remains for the identification of better diagnostic and monitoring AS biomarkers.

In this study, we investigated an extended aptamer-based panel of over 1300 proteins in biologic pharmacotherapy naïve AS patients to (1) discriminate AS patients versus healthy controls (HC) and (2) compare AS patients with active versus inactive disease.

## Methods and datasets

### Patient and specimens

Serum samples from the Prospective Study of Outcome in Ankylosing Spondylitis (PSOAS) cohort UTHealth biorepository were examined [16]. This multicenter cohort was initiated in 2003 and includes patients from UTHealth Houston, University of California San Francisco, the NIH Clinical Center, Cedars-Sinai Medical Center, and the Queensland University of Technology (Australia). The research carried out followed the Helsinki Declaration, each institution had the study approved by their respective institutional review boards (IRB), and each participating patient reviewed and signed an informed consent form. We studied age-, gender-, and race-matched patient and control sera in a 1:1:1 ratio for active, inactive, and healthy controls. All AS patients met modified New York classification criteria. Active disease was defined by an Ankylosing Spondylitis Disease Activity Score C-reactive Protein (ASDAS-CRP) of ≥ 2.1 and inactive disease as ASDAS-CRP of < 1.3 [17]. All patients were biologic and synthetic disease-modifying-anti-rheumatic drug (DMARD) naïve at the time of sera draw. Serum samples were immediately stored at a temperature lower than − 70 °C and had not been previously thawed.

### Serum protein determination

Serum samples were analyzed using the SOMAscan™ assay (SomaLogic; Boulder, CO), which is a sensitive and quantitative protein biomarker discovery platform. SOMAmers (Slow Off-rate Modified Aptamers), single-stranded DNA aptamers with modified nucleotides, bind to specific proteins in the serum that are then quantified as DNA [18, 19]. The SOMAscan assay quantified a total of 1320 proteins in each patient sample. Sample data was first normalized to remove hybridization variation within a run followed by median normalization across all samples to remove plate effects between runs. All plates were matched between active disease, inactive disease, and healthy controls in a 1:1:1 ratio, respectively. The median lower limit of quantitation for all measured proteins was 0.3 picomolar (pM), with a dynamic range of > 5 logs, and a median coefficient of variation (%CV) of 5%.

### Statistical analysis

*T*-tests and Mann–Whitney *U* tests were performed for active and inactive AS patients compared to healthy controls (diagnosis analysis) and active compared to inactive disease activity (Monitoring Analysis) in a 2:1 and 1:1 ratio, respectively. In our analyses, *p*-values and *q*-values (false discovery rate [FDR] corrected *p*-value) were calculated for all proteins using the Benjamini and Hochberg method in the R environment for statistical computing (http://www.r-project.org/). All analyzed proteins with a *p* < 0.05 were considered differentially expressed proteins (DEPs).

We used the Search Tool for the Retrieval of Interacting Gene/Proteins (STRING, https://string-db.org) database to analyze DEPs in our diagnosis and monitoring analyses for protein–protein interactions (PPI) with Cytoscape software. The Cytoscape Molecular Complex Detection (MCODE) plugin was used to find clusters in PPI networks, confidence cutoff of 0.4.

DEPs were also analyzed using Ingenuity Pathway Analysis (IPA) software (Qiagen https://digitalinsights.qiagen.com/products-overview/disovery-insights-portfolio/analysis-and-visualization/qiagen-ipa/) to identify active molecular targets. The goal of Upstream Regulator Analysis is to identify upstream molecular regulators and to predict whether they are active or inhibited. This analysis is based on expected causal effects between upstream molecular targets. A *Z*-score algorithm is used to make predictions with *Z*-scores > 2 and <  − 2 considered significant.

A multi-biomarker panel among our DEPs for diagnosis were selected using a L1-penalized logistic regression using the least absolute shrinkage and selection operator (lasso) classifier, over a range of lambda, a tuning parameter that determines how many biomarkers are selected. Lasso regression analysis was performed of our diagnosis DEPs to determine optimal protein combination in a 2:1 training/test split. The models were evaluated using cross-validation and inspecting plots receiver operating characteristic (ROC) curves. R packages, *glmnet* and *caret*, were used for these statistical analyses.

## Results

### Demographic and clinical characteristics

We studied 80 study patients’ sera, *n* = 26:26:28 for active, inactive, and HC, respectively. Study patients’ sex, age, and other important clinical characteristics are summarized in Table 1. Our AS patients were a mean 46 ± 14 years of age with a symptom duration of average of 25 ± 13 years. All patients and controls were White.

**Table 1:** Demographic and clinical characteristics of the PSOAS participants and control subjects

Characteristic	High AS disease activity (*N* = 26)	Low AS disease activity (*N* = 26)	Healthy controls (*N* = 28)
Age (mean, standard deviation (SD); years)	46.08 ± 13.58	45.48 ± 12.73	44.44 ± 15.85
Male gender (*n*, %)	16 (61%)	16 (61%)	17 (61%)
AS symptom duration (mean SD; years)	25.47 ± 13.32	25.16 ± 12.82	–-
ASDAS-CRP^a^ (mean SD)	3.68 ± 0.67	0.40 ± 0.37	–-
HLA-B27 positivity (*n*, %)	19 (73%)	24 (92%)	–-
Met Modified New York Criteria	26 (100%)	26 (100%)	–-

^a^Ankylosing Spondylitis Disease Activity Score C-reactive Protein

### Serum protein levels

**Fig. 1:**
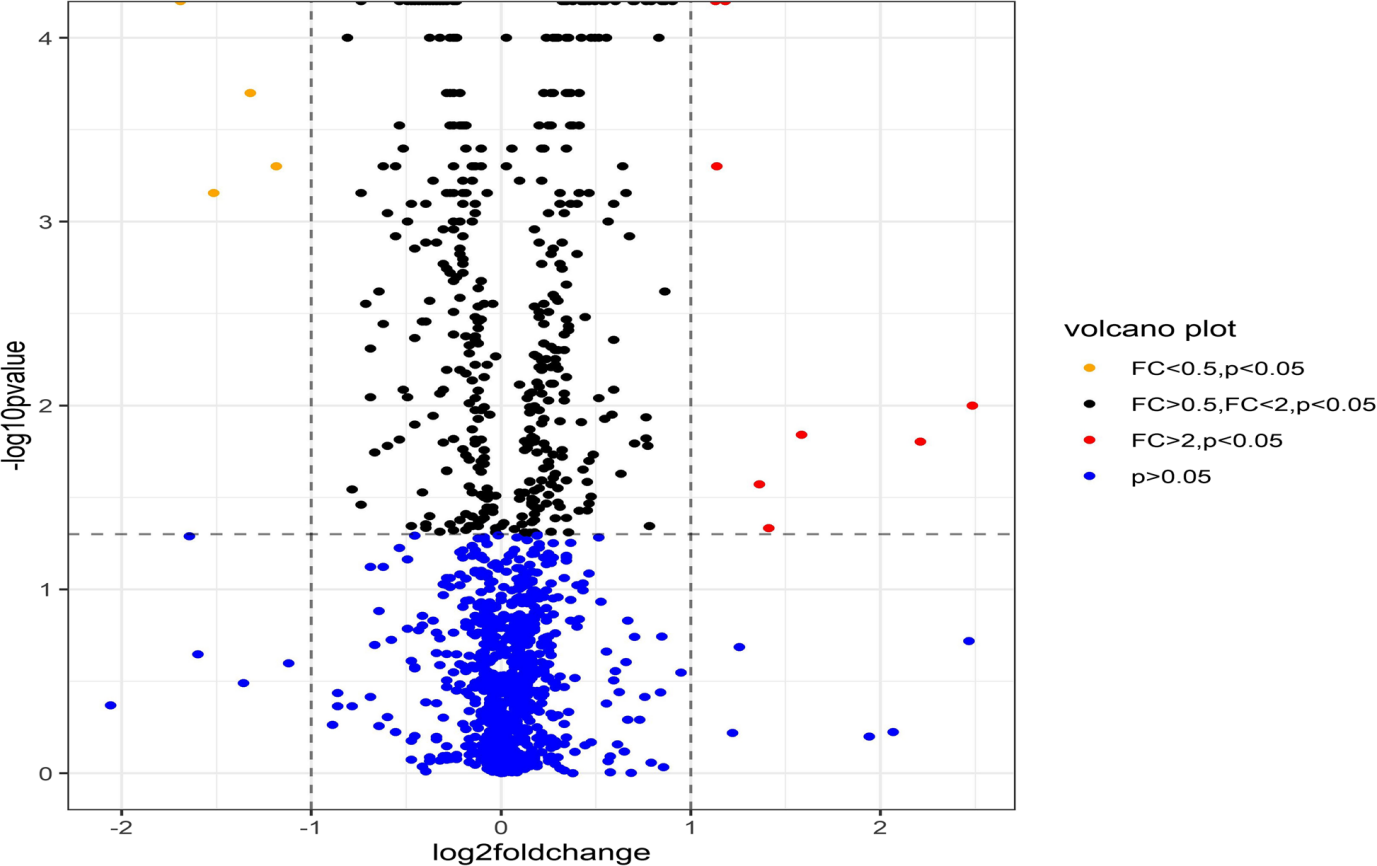
Volcano plot of all serum proteins for **A** diagnosis (AS patients vs. healthy controls) and **B** monitoring (AS high vs. low disease activity patients). Fold change (FC) of AS patients compared to controls < 0.5, 0.5–2.0, and > 2 is presented in yellow, black, and red, respectively. 367 and 157 proteins were differentially expressed for diagnosis and monitoring, respectively

Of the 1317 proteins detected in our diagnosis and monitoring analyses, 367 and 167 DEPs were detected, respectively (*p* < 0.05) (Fig. 1). When corrected for multiple comparison (FDR-corrected *q* < 0.05), 247 and 27 DEPs remained for diagnosis and monitoring, respectively. Thirteen DEPs overlapped between our diagnosis and monitoring analyses (Table 2). Eleven of the 13 overlapping DEPs had associations in concordant direction for diagnosis and monitoring. The top 10 upregulated and downregulated DEPs based on fold change for diagnostic and monitoring biomarkers are presented in Table 3. The complete list of differentially expressed proteins is available in Supplementary Table 1.

**Table 2:** Shared AS diagnostic and monitoring serum proteins

Protein name	Fold change (active vs inactive)^a^	*q*-value^b^	Fold change (AS vs HC)^a^	*q*-value^b^
Testican-2	0.81	0.02	1.23	< 0.01
Factor I^c^	1.22	< 0.01	1.13	< 0.01
FCN1	1.24	< 0.01	0.91	< 0.04
C6^**c**^	1.26	0.02	1.20	< 0.01
C5b, 6 Complex^c^	1.26	0.01	1.15	0.02
Factor B^**c**^	1.33	< 0.01	1.24	< 0.01
LBP^c^	1.48	< 0.01	1.23	< 0.02
C9^c^	1.53	< 0.01	1.33	< 0.01
Haptoglobin, Mixed Type^c^	1.56	0.04	1.51	< 0.01
FUT5^c^	1.65	0.04	1.73	< 0.01
C5a^c^	1.92	< 0.01	1.87	< 0.01
CRP^c^	3.33	< 0.01	2.20	< 0.01
SAA^c^	10.65	< 0.01	5.60	< 0.01

*Abbreviations: Factor I* Complement Factor I, *FCN1* Ficolin-1, *C6* Complement Component 6, *C5b 6 Complex*, Complement Component 5b, 6 Complex c, *Factor B* Complement Factor B, *LBP* lipopolysaccharide binding protein, *C9* Complement Component 9, *FUT5* Fucosyltransferase 5, *C5a* Complement Component C5a, *CRP* C-reactive protein, *SAA* serum amyloid A

^a^Values of > 1 refer to upregulated expression of proteins and values of < 1 refer to downregulated expression of proteins in ankylosing spondylitis

^b^False discovery rate-corrected *p*-value for multiple testing

^c^Concordant fold change for diagnosis and monitoring

**Table 3:** Top upregulated and downregulated serum proteins detected by a 1320-plex proteomic screen

Diagnostic biomarkers (AS vs. healthy control)				Monitoring biomarkers (high vs. low disease activity)			
**Protein name**	UniProt ID	**Fold change** ^a^	***q*** **-value** ^b^	**Protein name**	UniProt ID	**Fold change** ^a^	***q*** **-value** ^b^
C4b	P0C0L4 P0C0L5	0.31	< 0.01	Hemoglobin	P69905, P68871	0.14	< 0.01
17-beta-HSD 1	P14061	0.35	0.01	CK-MB	P12277 P06732	0.34	0.01
Phosphoglycerate mutase 1	P18669	0.40	< 0.01	Cathepsin V	O60911	0.59	< 0.01
TF	P13726	0.44	< 0.01	Kallistatin	P29622	0.70	< 0.01
PTP-1C	P29350	0.57	< 0.01	CD36 ANTIGEN	P16671	0.70	< 0.01
H2B2E	Q16778	0.57	< 0.01	CNDP1	Q96KN2	0.72	0.02
PRKACA	P17612	0.60	< 0.01	aldolase A	P04075	0.74	0.01
Lactoferrin	P02788	0.60	0.01	NCAM-120	P13591	0.77	0.04
CAPG	P40121	0.61	0.02	Catalase	P04040	0.77	0.04
Azurocidin	P20160	0.62	0.03	Glypican 3	P51654	0.77	0.03
FUT5	Q11128	1.73	< 0.01	FUT5	Q11128	1.65	0.01
PAPPA	Q13219	1.78	< 0.01	Fibrinogen	P02671 P02675 P02679	1.90	0.01
SARP-2	Q8N474	1.80	< 0.01	C5a	P01031	1.92	< 0.01
MMP-12	P39900	1.82	0.01	HGF	P14210	1.99	0.02
Glucagon	P01275	1.82	< 0.01	C3b	P01024	2.26	< 0.01
C5a	P01031	1.87	< 0.01	NPS-PLA2	P14555	2.39	0.02
TFF1	P04155	2.19	< 0.01	CRP	P02741	3.33	< 0.01
CRP	P02741	2.20	< 0.01	Fibrinogen g-chain dimer	P02679	4.32	< 0.01
6-Phosphogluconate dehydrogenase	P52209	2.27	< 0.01	D-dimer	P02671 P02675 P02679	8.38	< 0.01
SAA	P0DJI8	5.60	0.04	SAA	P0DJI8	10.65	< 0.01

*Abbreviations: C4b* Complement Component 4b, *17-beta-HSD 1* 17β-Hydroxysteroid dehydrogenase 1, *TF* Tissue Factor, *PTP-1C* Tyrosine-protein phosphatase non-receptor type 6, *H2B2E* Histone H2B type 2-E, *PRKACA* cAMP-dependent protein kinase catalytic subunit alpha, *CAPG* Macrophage-capping protein, *FUT5* Fucosyltransferase 5, *PAPPA* Pappalysin-1, *SARP-2* Secreted frizzled-related protein 1, *MMP-12* Macrophage metalloelastase, *C5a* Complement Component 5a, *TFF1* Trefoil factor 1, *CRP* C-reactive protein, *SAA* serum amyloid A, *CKMB* Creatinine Kinase M-type, *CNDP1* Beta-Ala-His dipeptidase, *NCAM-120* neural cell adhesion molecule 1, *HGF* hepatocyte growth factor, *NPS-PLA2* Phospholipase A2, membrane associated

^a^Values of > 1 refer to upregulated expression of proteins and values of < 1 refer to downregulated expression of proteins in ankylosing spondylitis

^b^False discovery rate-corrected *p*-value for multiple testing

### Pathway analyses

**Fig. 2:**
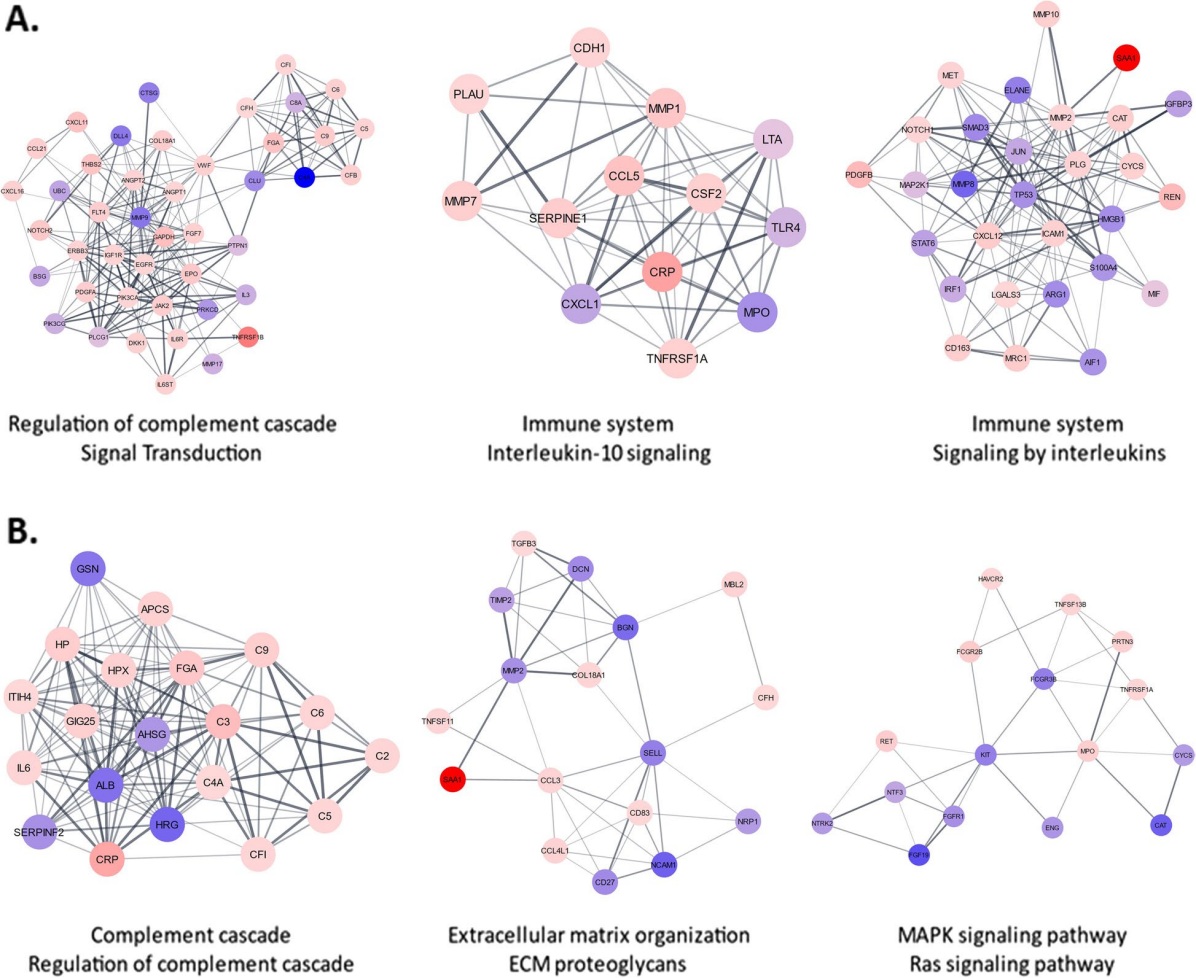
Protein–protein interaction networks. **A** Top 367 diagnosis DEPs and **B** top 157 monitoring DEPs (*T*-test *p* < 0.05) through the CytoscapeSTRING App with a confidence cutoff of 0.4. Molecular Complex Detection (MCODE) clustering was performed and displayed are the top three clusters. The color of each MCODE node corresponds to the fold change. Nodes with a fold change less than one range in color from blue to purple while those with a fold change greater than one range from pink to red. The confidence score of each interaction is displayed as the edge thickness and opacity. The top two reactome pathways associated with each cluster are displayed below each cluster

MCODE analyses identified complement regulation/signal transduction, interleukin (IL)-10 signaling/immune system, and immune system/interleukin signaling as the top 3 overrepresented pathways. Complement, extracellular matrix organization/proteoglycans, and mitogen-activated protein kinase(MAPK)/rat sarcoma virus protein (RAS) signaling were the top 3 overrepresented pathways among the monitoring biomarkers (Fig. 2).

**Fig. 3:**
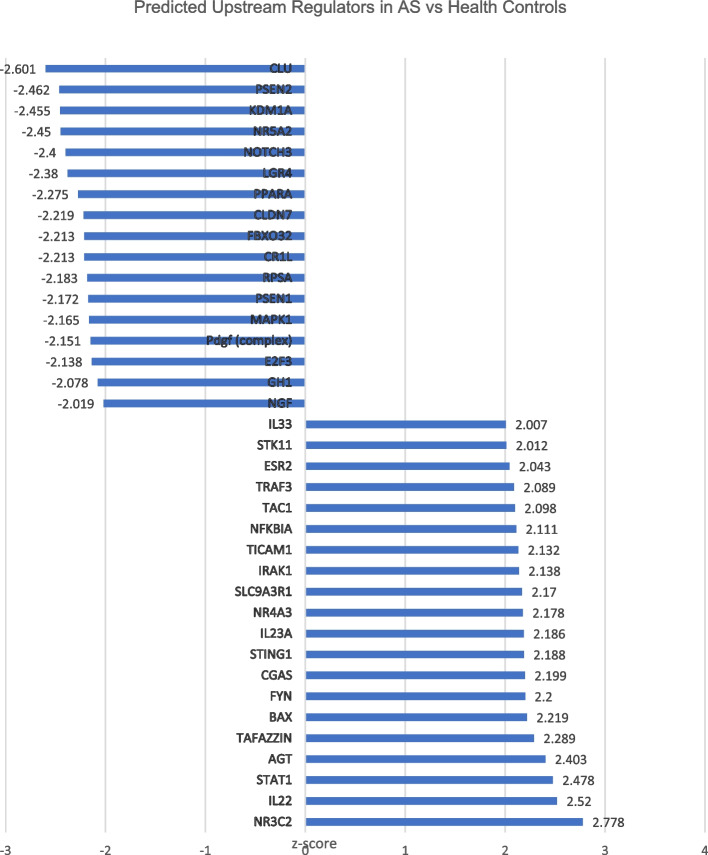
Top predicted upstream molecular regulators based on the Ingenuity Knowledge Base. *X*-axis shows the activation *Z* score calculated based on the Ingenuity Pathway Analysis for identifying upstream regulators. Proteins that were differentially expressed with ankylosing spondylitis compared to healthy control subjects used were input into Ingenuity

The IPA upstream analysis predicted, activated upstream regulators included IL 23/17 (IL-22, IL-23A), tumor necrosis factor (TNF receptor-associated factor 3), cGAS-STING (cyclic GMP-AMP synthase, Stimulator of Interferon Gene 1), and Jak/Stat (Janus Kinase/Signal transducer and activator of transcription 1), signaling in AS. Predicted inhibited upstream regulators included those involved in lipid metabolism (Nuclear Receptor Subfamily 5 Group A Member 2, Peroxisome proliferator-activated receptor alpha) and protein folding (Clusterin, Presenilin-2) (Fig. 3.)

### Lasso regression

**Fig. 4:**
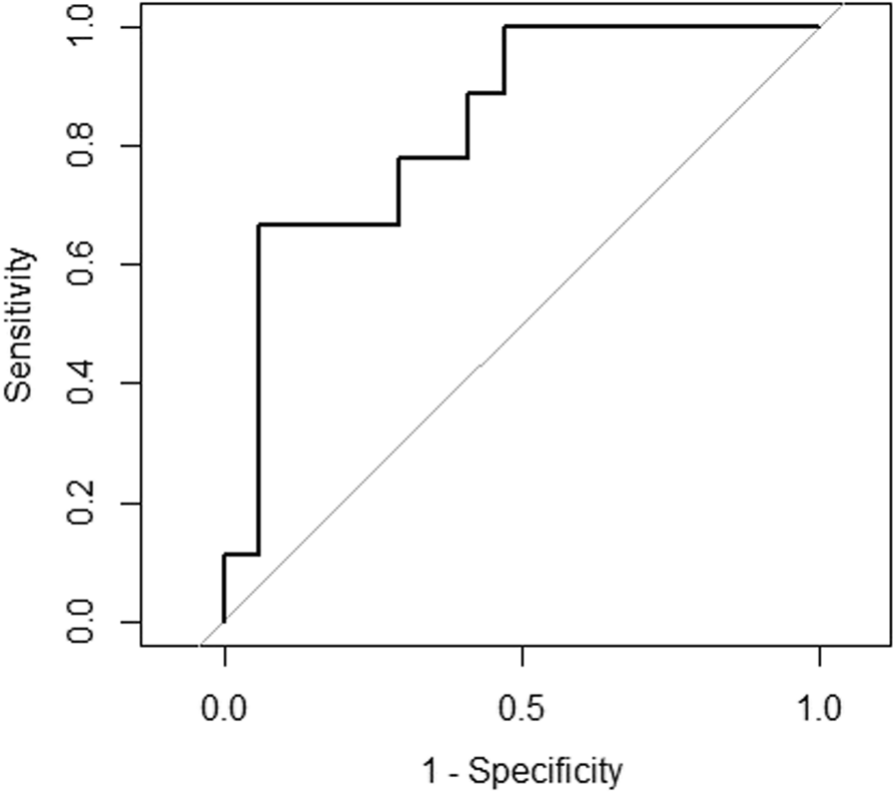
Receiver operator curve of diagnostic 13-protein LASSO discrimination of AS patients vs. controls

All samples were randomly assigned in a 2:1 ratio between discovery (*N* = 54) and validation sets (*N* = 26). Lasso regression identified a Diagnostic 13-DEP model predictive of AS: Immunoglobulin A (IgA), Complement component 5a (C5a), Secreted frizzled-related protein 1 (SARP-2), Secretory leukocyte peptidase inhibitor (SLPI), Cathepsin A (CTSA), Neurexophilin 1 (NXPH1), C-X-C motif chemokine ligand 16 (CXCL16), Interleukin 6 signal transducer (gp130), complement component 4b (C4b), Cofilin-1 (CFL1), Cell adhesion molecule L1 like (CHL1), Signaling lymphocytic activation molecule family member 6 (SLAF6), and Macrophage mannose receptor (MRC1). This model had a sensitivity of 0.75, specificity of 0.90, kappa of 0.59, and an overall accuracy of 0.80 (95% CI: 0.61–0.92). The predictive probability of our model to discriminate AS patients vs controls based on ROC curve (95% CI) was 0.79 (0.61–0.96) (Fig. 4). The McNemar’s test of our model was non-significant (*p* > 0.05), suggesting a similar proportion of misclassification for diagnosis (e.g., false negatives and false positives). We did not perform Lasso regression for monitoring as this would have not been meaningful due to the modest sample size.

## Discussion

The goal of this study was to identify candidate biomarkers for AS diagnosis and monitoring disease activity. Our proteomic screen of ankylosing spondylitis identified 317 and 59, FDR-corrected, DEPs for diagnosing and monitoring, respectively. Further bioinformatics tools highlighted signaling pathways and potential *in silica* upstream regulators. In our PPI analyses, we elucidated complement and immune signaling importance in diagnosis and MAPK/RAS for monitoring disease activity. IPA upstream analysis predicted activated TNF, IL-23/17, and cGAS-STING signaling pathway targets as well as inhibited lipid metabolism and protein folding targets. Finally, our machine-learning model showed that a 13-DEP model had ability to discriminate AS patients from controls with modest discrimination, AUC 0.79.

These results add to our knowledge of ankylosing spondylitis, showing that serum proteins can molecularly distinguish AS from healthy controls. We also elucidated additional proteins that differentiate between disease activity states that may be useful in monitoring disease in clinic. Bioinformatics tools highlighted important inflammatory and immunologic pathways important for AS pathogenesis. This including well-known AS association of TNF, IL 23/17, and JAK/Stat signaling that contrasted with cGAS-STING, MAPK/RAS signaling that were identified as well.

Previous biomarker studies in AS have largely focused on individual proteins based on a priori knowledge of biomarkers in associated conditions [20]. Comprehensive discovery studies of AS biomarker susceptibility have only used mass spectrometry to date. In contrast, our study was based on broad screens of aptamer-based libraries of > 1300 proteins in an unbiased fashion. Our study is the first to use aptamer-based high-throughput technology to capture low-abundance serum proteins in AS patients and controls, identifying DEP new potential biomarkers for diagnosis and disease activity in addition to confirming previous findings. For example, among our top diagnosis DEPs, SAA1 has now been shown in multiple studies to be elevated in AS while Trefoil Factor (TFF)1 has not been reported in AS/AxSpA [10–12].

While the pathophysiologic function of TFF1 is unknown, TFF peptides modulate cell junctional complexes thus contributing to the gastrointestinal epithelial barrier function [21]. Impairment of the mucosal epithelial barrier is a hallmark of inflammatory bowel disease (IBD) [22]. TFF levels increase upon epithelial injury, presumably to prevent further damage and disease progression in IBD [23–25]. Beyond IBD, TFF1 is also upregulated in the intestine in response to injury [26, 27]. Given TFF1 long known association with inflammatory bowel disease, a condition seen in overlap with AS, and the large prevalence of colitis in AS, our newly identified DEPs may be useful AS biomarkers [24].

Among our 11 concordant biomarkers for diagnosis and monitoring, they can be grouped into three categories: acute phase reactants (e.g., SAA1, Haptoglobin, CRP), complement (e.g., C5a, C5b-C6 complex, C6, C9, Factor B, Factor I), and lipopolysaccharide-related proteins (LPS) (e.g., FUT5, LBP). SAA1 had greater fold difference than CRP, suggesting it may be a more useful biomarker than CRP for diagnosis and disease activity. Complement system proteins, most notably C4, have been reported to be upregulated in ankylosing spondylitis [28, 29]. It has been suggested that complement activation may be a key pathway involved in AS pathogenesis through murine studies [30]. We identified additional complement proteins in our study that may elucidate complement involvement in AS. Gram-negative bacteria are long thought to be an environmental trigger for ankylosing spondylitis, which relates to our LPS protein findings [31]. Furthermore, prior peripheral blood gene expression studies in AS have identified an upregulation of Toll-like receptor 4 (TLR4) AS which is the ligand of LBP and LPS [7, 32]. Our results support the involvement of LPS-LBP/TLR4 axis AS pathogenesis and disease activity.

We also piloted lasso regression, a machine-learning technique, to find a protein panel that can best identify AS patients. This led to a 13-protein panel with an overall AUC superior to that of C-reactive protein, the commonly used AS serum biomarker [33]. Our protein panel performance was comparable to previous reports of advanced imaging including MRI and low-dose CT [34]. These proteins included complement and interleukin-6 signaling proteins, already highlighted as important pathways as well as other inflammation-associated proteins. For example, SLAF6, also known as SLAMF6, has been shown to lead to increased IL-17 production in autoimmune conditions [35–43]. These candidate proteins require further investigation to determine their clinical utility singularly and in combination.

This project had limitations. While all patients studied were not exposed to biologic or synthetic DMARDs, non-steroidal anti-inflammatory drugs (NSAIDs) were not accounted for in our analyses. This treatment modality might have some impact on the serum protein profile; however, we expect that NSAIDs would lead to a decrease in the serum inflammatory markers. We instead observed an increase in the inflammatory serum proteins in the AS versus control comparison, suggesting that the observed molecular profile is disease-related rather than secondary to NSAID treatment. Most of our AS patients had long-standing disease; serum biomarkers may differ between early and established AS. The large number of potential biomarkers and modest sample size of patients may make our findings susceptible for overfitting. We chose Lasso modeling over other machine-learning techniques due to the penalization limiting this potential error. We also chose to study ankylosing spondylitis patients compared to healthy controls, two distinct conditions. Biomarkers that could distinguish non-radiographic axial spondyloarthritis compared to non-specific chronic lower back pain would have greater clinical utility. Our results thus can only be taken in an indirect context for the aforementioned clinical scenario. Biomarkers identified thus in our study require further testing in patients with the full AxSpA spectrum compared to various chronic lower back pain conditions that mirror clinical symptoms of AxSpA.

The clinical utility of our identified biomarkers requires replication. Our findings indicate that the required sample size will differ for each DEP. For example, serum amyloid A requires only 35 patients per group (diagnosis) while Complement Factor I would require 116 patient samples (diagnosis) for replication as diagnostic biomarker based on their delta/standard deviation from our proteomic screen at a power of 80% and significance of 5%. Our study however adds to the literature of potential candidate biomarkers that can aid in AS diagnosis and monitoring of disease activity.

## Conclusions

In summary, the current methods for the diagnosis of AS patients have relied on a combination of patient-reported symptoms, imaging, and non-specific acute phase reactants [17]. These have been useful for the classification to study disease; however, among the nearly 20% of the US population with chronic back pain, there remains a large portion of potentially undiagnosed AxSpA. Better biomarkers would address a significant unmet need both in clinic, where earlier diagnosis and referral might be improved, as well as in bettering our understanding of disease mechanisms [44]. Our study adds to the current literature by highlighting inflammatory pathways involved in AS diagnosis and disease activity as well as using machine learning to identify potential diagnostic and monitoring biomarkers in AS. This project may serve as a useful adjunct to earlier diagnosis, more accurate disease activity monitoring, and development of a predictive prognosis model that can ultimately lead to earlier AS diagnosis and treatment.

## Supplementary Information

**Additional file 1:Supplementary Table 1.**

## Data Availability

The datasets used and/or analyzed during the current study are available from the corresponding author on reasonable request.

## Abbreviations

AS: Ankylosing spondylitis
AxSpA: Axial spondyloarthritis
C4b: Complement component 4b
C5a: Complement component 5a
CFL1: Co-filin-1
cGAS-STING: Cyclic GMP-AMP synthase, Stimulator of Interferon Gene 1
CHL1: Cell adhesion molecular L1 like
CRP: C reactive protein
CXCL 16: C-X-C motif chemokine ligand 16
CTSA: Cathepsin A
DEPs: Differentially expressed proteins
DMARD: Disease-modifying anti-rheumatic drug
ESR: Erythrocyte sedimentation rate
FDR: False discovery rate
Gp130: Interleukin 6 signal transducer
IBD: Inflammatory bowel disease
IgA: Immunoglobulin A
IL: Interleukin
IPA: Ingenuity Pathway Analysis
Jak-Stat: Janus Kinase-Signal transducer and activator of transcription 1
Lasso: Least absolute shrinkage and selection operator
LPS: Lipopolysaccharide
MAPK: Mitogen-activated protein kinase
MCODE: Cytoscape’s Molecular Complex Detection plugin
MRC1: Macrophage mannose receptor
NXPH1: Neurexophilin 1
NSAIDs: Non-steroidal anti-inflammatory drugs
PPI: Protein-protein interactions
RAS: Rat Sarcoma Virus Proteins
ROC: Receiver operating characteristic
SAA1: Serum amyloid A1
SARP-2: Secreted frizzled-related protein 1
SLPI: Secretory leukocyte peptidase inhibitor
SOMA: Slow Off-rate Modified Aptamers
STRING: Search Tool for the Retrieval of Interacting Genes/Proteins
TLR4: Toll-like receptor 4
TFF: Trefoil Factor
